# Notes on Medical Matters and Medical Men in Paris

**Published:** 1848-01

**Authors:** David W. Yandell

**Affiliations:** Louisville, Ky.; Paris


### THE WESTERN JOURNAL OF MEDICINE AND SURGERY.

JANUARY, 1848.

ART. I.—*Notes on Medical Matters and Medical Men in Paris.* By DAVID W. YANDELL, M.D., of Louisville, Ky.

There are few wards in Paris that offer more instructive cases to the student than those of M. Jobert, at Saint Louis. The following is one which he treated not long since. The case was one of *spontaneous gangrene* occupying the left foot of a man aged thirty-nine years, a painter by occupation. Here, as is almost always the fact, the cause of the disease remains unknown. It is true, as we shall see further along, that an arteritis had preceded the appearance of the gangrene; but the difficulty is not thus removed, and, besides, nothing reveals under what influence the arteries of the leg became inflamed. In fact, remarked M. J., our patient is of a very good constitution; he has never committed excesses of any kind, and he assures us that no mechanical violence was concerned in the production of the disease. It is now seven years since he commenced to feel in the leg pains which appeared at intervals for four years, when they became continued, and augmented in intensity; in the last two years he has remarked that his leg, while the whole of it became cold, diminished in volume. Finally, about six weeks since, the foot blackened, and the skin which enveloped it became covered with numerous phlyctænæ. At the time of the operation, you will remark the following condition of things: blackish tint of the toes, upon which the skin is dry and horn like; coloration of the same nature on the back of the foot, with this difference, that the gangrene there is moist rather than dry; in front of the tibio-tarsal articulation a large eschar, with a greyish black base, and the discharge of an ichorous liquid, testifies the presence of a recent mortification of the skin of this region. A little above this wound, the skin is red around the whole circumference of the leg, and this redness, which terminates abruptly, has remained stationary for some days, indicating that the gangrene is not in a stage of progression. Except the discoloration, the dermis is in all respects in a state of integrity. The patient was carried to the amphitheatre, and after inhaling the vapors of ether for some minutes, M. Jobert removed his leg according to the flap method, at the middle third. The arteries furnished very little blood, and the operator remarked that the blood was blacker than in the normal state, and that, consequently, there had been a commencement of asphyxia. The patient, who at the moment of the division of the skin had moved suddenly, preserved no recollection of the pain. It was easily to be seen, after the lapse of a few days, that not only re-union by first intention would not be effected, but that, unfortunately, the gangrene continued its ascending march; soon, in fact, the soft parts fell in eschars and the denuded bones projected beyond them. A continued febrile action, delirium, general debility, and the appearance of a large gangrenous eschar upon the sacrum, announced a speedily fatal termination, and death occurred on the twelfth day after the operation.

The foot and the inferior part of the leg having been dissected, M. Jobert pointed out the obliteration of the tibial arteries, which bore traces of a recent inflammation.

*M. Jobert's Method of Treating Fractures.* For the last fourteen years, M. Jobert has treated fractures of the leg, I may say, without the bandage. It would be strange indeed if a man of his intelligence, who in more than one respect is in advance of his countrymen, should persist in any plan of treatment, for so many years, if it was not at least equal, and on some accounts superior, to the more common, and, as I might say, more orthodox systems. Placing the fractured member upon a cushion, he embraces the foot with a simple bandage, in the form of a stirrup, the ends of which serve to effect extension, which is afterwards rendered permanent by their being firmly attached to the foot of the bed. Counter-extension is practised by means of a towel, the centre of which is placed upon the pelvis, and the two extremities fastened to the head of the bed. In this way the limb is ready for the application of topical remedies if rendered necessary, either by a wound of the integuments, a phlegmon, or any other affection. This, however, according to Jobert, is not the only advantage of this method of treatment. The fractured limb, not being submitted to any compression, its circulation is performed as in the normal state; and certainly it cannot be denied that this is a condition in every way favorable to the prompt formation of the callus. Moreover, in the leg, the muscular powers act but little, and a feeble force is sufficient to maintain the osseous fragments in a position suitable for obtaining a consolidation without shortening.

I do not propose to compare this mode of treatment of fractures of the leg with those employed by other surgeons. It is at any rate simple, and any one who will follow M. Jobert in his visits will see, among probably the largest number of fractures that are to be found in any service in Paris, the most perfect and beautiful cures of this class of accidents effected by a strip of bandage and a towel.

*Burns.* Dupuytren laid down six degrees of burns, according to their intensity, a division than which there is probably not a better. His classification was the following: 1st, superficial inflammation of the skin without phlyctens; 2d, inflammation with phlyctens; 3d, disorganization of a portion of the papillæ of the skin; 4th, the dermis reduced to eschar; 5th, combustion of the tissues to the bone; 6th, carbonization of the whole of a member. M. Jobert is in the habit of treating burns by the application of bladders filled with ice, the part being previously covered by a compress with holes punched in it and a large anodyne of cerate spread upon it. M. J. is of opinion that recovery is more promptly obtained in this way. On this subject I do not pronounce an opinion, but there is no doubt that the pain, which is the principal feature in burns of the skin, is relieved in an astonishing degree from the first hour of the application of the ice.

The following cases treated a short time since by M. M. Jobert and Malgaigne, in the wards of St. Louis, afford an illustration of their modes of treatment:

In consequence of the explosion of a steam boiler, sixteen men, who were occupied around it, were burned and otherwise injured, four of whom died immediately; the remaining twelve were carried to St. Louis, nine into the wards of Malgaigne, three into those of Jobert. The treatment of these two surgeons being different, I will briefly notice the condition and management of the whole, commencing with the nine in Malgaigne's ward.

Case I.—*Wound of the Head, with loss of substance of the Bone and Encephalon.—Death.* In bed No. 30 was placed a man named Bernard æt. twenty-one years. His body and extremities were covered with contusions and burns; the superior part of his head presented a vast wound with loss of substance of the bones and encephalon. Besides this, the skin was cold; pulse extremely feeble, scarcely perceptible; complete loss of sensibility and consciousness; stertorous respiration. Malgaigne, after carefully examining the unfortunate man declared that there was no chance of his recovery. This was at eleven o'clock, A. M.; at half-past one o'clock, P. M., he died.

CASE II.—*Grave Burn of the Face, Trunk, and Superior Extremities.* The patient, a man twenty-three years old, presented a deep burn of the whole right half of the face, the forearms and the hands, and of the right side of the chest. He suffered the most distressing pains. Pulse 112, small, hardly perceptible. On the day of his entry a very abundant discharge of blood occurred from the auditory channel of the right side, which ceased the following day. Malgaigne prescribed on the 7th (the day of entry) an infusion of tilia (*tilia Europea*), and on the 8th a honied injection. (*Lavement miellé.*) The burns were dressed with the lime liniment and cotton batting. Five days elapsed, and although the patient had outlived the pain, there was one of the burns which created the greatest anxiety, in view of the reaction which precedes the fall of the eschars and the suppuration which necessarily accompanies it. On the 11th the work of elimination commenced. The pulse was very hard and frequent, 120; skin hot; thirst intense. The patient frequently endeavored to get up from bed, and was obliged to be constantly watched. At one o'clock, A. M., on the 12th, he died.

CASE III.—*Burns of the Trunk and Members; Wounds of the Face and Scalp.* Joseph Chopin, æt. twenty-five years, offered a burn of the chest, thighs and right arm, wounds upon the scalp, nose and right eye-lid which presents considerable ecchymosis. Malgaigne prescribed the infusion of tilia for tisane, and dressing with the lime liniment and cotton. At the evening visit, the pulse which in the morning was feeble and at 64, regained its force and frequency, 92. The interne took about twelve ounces of blood from the patient's arm. On the 8th, Malgaigne, finding the pulse as frequent though less strong. did not repeat the bleeding. But on the 10th, the pulse having again augmented in force and frequency, the patient was bled both in the morning and evening. On the 11th, the patient was in almost the same condition, no accident having yet supervened. On the 13th, an intense erysipelas was developed on the face and scalp, and at ten o'clock, P., M., on the 15th, he died.

CASE IV.—*Burn of the Buttock and the Right Superior Extremity.—Contusions of the Face.* Renard, æt. fifty-six years, presented a very deep burn about the size of the hand, on the right buttock, also another situated on the right arm about the humero-cubital articulation. There were also contusions upon the face. In the morning the expression of the patient's face was somewhat stupified; his pulse small and at 112. During the day the pulse returned to almost the normal condition at 64 pulsations, but in the evening it regained in force and frequency; it was then 84. Venesection. On the 8th, the patient said he had slept the night previous; he then appeared a little drowsy, was pretty well and asked for something to eat. Skin moist, pulse 80. Ordered sugared gum; some dressing, broths and soups. On the 11th, the patient complained of pain from his burn on the buttock only when lying upon his back, and again asked for something to eat.

CASE V.—*Contusion of the Head and of the Right Leg.* Victor, aged seventeen years, who had been more scared than hurt, only offered some slight contusion on his head and right leg. On the next day, being able to go about, he quit the hospital.

CASE VI.—*Burn of the Right Buttock.—Wounds of the Head and Right Thigh.* Joseph, æt. twenty-six years, presented on the right buttock a superficial burn not larger than the hand, and on the scalp a small wound without denudation of the bones. A wound that was not at all serious was observed also upon the right thigh. The evening of the day of the accident the patient's skin being hot, pulse hard and frequent, the interne bled him. On the 8th, Malgaigne ordered the wound to be dressed with the oleo-calcarious liniment and carded cotton. The pulse, although still frequent, (112) had lost the force which it possessed the preceding day, which circumstance induced M. not to prescribe another bleeding. In other respects the condition of the patient was so satisfactory that Malgaigne did not hesitate to give him broths. The following day no accident supervened. On the 10th, the patient was able to eat a portion of solid food, and on the 11th he was allowed two. On the 16th, no complication has yet occurred; the wound of the thigh suppurates a good deal, and there exists a circumscribed swelling and pain around it.

Case VII.—*Contusions of the Inferior Members.—Wounds of the Head and Right Hand.* Betbeze, æt. twenty-five years, who was one of those who escaped the most fortunately, presented only two wounds, neither of which was serious, the one superficial and of small extent, situated on the scalp, the other on the right hand. Some traces of contusions are to be seen on the inferior extremities. There did not supervene in this case any reaction, nor any of those accidents which are so often observed in consequence of wounds of the head. On the 11th, the patient eat two portions of aliment, and walked in the garden. On the 16th, Malgaigne signed his *exeat.*

Case VIII.—*Wound of the Head.—Deep Burn of both Buttocks, and of the Left Leg.* Rolland was standing beside case No. 2 when the boiler burst, and although he suffered considerably, he was less mutilated than his neighbor; he presented nevertheless a deep burn of both buttocks, and two wounds, the one on the head and the other on the neck. The superior right eye-lid was the seat of a very considerable sanguine effusion. On the 8th, notwithstanding these lesions, Malgaigne found the patient in a state sufficiently satisfactory to warrant him in allowing him to take two soups. The burns were dressed with the oleo-calcarious liniment and the wound covered with simple dressings. On the 11th, no complication had supervened, and the patient eat a portion of aliment. On the 16th, the eschars commenced to detach themselves, and the suppuration was very abundant. Nevertheless the general condition of the patient was very satisfactory; he eat two portions.

Case IX.—*Wound of the Head.* Louis, æt. twenty-seven years, offered only a wound of the head, which caused some reactional symptoms that disappeared after two bleedings.

On the 11th, the patient commenced to eat, and in a few days was entirely restored.

Case X.—*Burn of the Thigh and Right Buttock.—Wound of the Left Leg.* Peter, æt. thirty-four years, presented a wound on the right buttock, and the superior part of the right thigh. There was observed also on the internal and middle part of the left leg a wound of a round form, of the size of a five franc piece, from which the muscles of the calf, violently contused and torn, project. Finally, the inferior members, and especially the right thigh, were more or less seriously contused. The patient, whose face bears the stamp of stupor, suffers acute pains. The pulse is frequent, but feeble. In the evening the stupor had disappeared, and the pulse had regained its force, and become still more frequent. Ordered bleeding. On the 8th, M. Jobert found the patient in the following condition: Skin hot, pulse hard and frequent. Sleepless night because of the severe pains which he had felt in his leg. Around the wound the skin was tense and red; the portion of the muscles which form a hernia beyond the edges of the wound are of a greyish color. The surgeon enlarged the wound by making, immediately above and below, two incisions, in order to relieve the strangulation, and thus prevent the mortification of the tissues. Being unable, from their situation, to apply ice to the burns, M. Jobert ordered them to be covered with simple dressing, and prescribed veal broth, sedlitz, bleeding, diet. On the 11th, the pulse was still full and frequent, and the skin hot but not dry. The large incisions made by the surgeon have not sufficed to prevent the mortification of the tissues which had been violently contused; and the leg presented on its internal surface, and in almost its whole length, a sphacelus which comprehended the skin and more or less of the subjacent tissues. On the 13th, the condition of the patient was in every way satisfactory, and he ate daily his allowance of food.

Case XI.—*Wounds of the Head and Left Foot.*—Toussaint, æt. forty-eight years, presented on the middle of the forehead an extensive wound, which was prolonged for some distance upon the scalp. The superior eye-lid of the left side offered considerable ecchymosis. There was also a small and unimportant wound of the inferior part of the left leg. The patient had at no moment lost his consciousness; he did not complain a great deal of pain, though he spoke of a sensation of heaviness in the head. At the evening visit his pulse was hard and frequent. Ordered bleeding. On the 8th, and the day following, Jobert prescribed emeticised veal broth; venesection; cataplasms; diet. On the 13th, after five copious bleedings, no accident has supervened; the sanguine effusion in the eye-lid has been absorbed: the case was progressing well; allowed him a portion of food. On the 16th, the state of the patient continued to be satisfactory.

Case XII.—*Burn of both Hands and of the Right Knee.* Mennier, æt. forty-six years, was burnt on the dorsal surface of the two hands, and also upon the superior part of the right knee. On the 8th, Jobert ordered the burns to be covered with a cloth dressed with cerate, and upon this bladders to be laid filled with ice; he prescribed, at the same time, emeticised veal broth; bathing with warm water; diet. On the 10th, no complication had occurred; allowed a portion of food. On the 13th, the patient complained of pain in the left side of the chest; but the most careful examination revealed nothing. Notwithstanding this, however, Jobert ordered some cups to be applied to the painful part. 16th, patient continued to progress well. The pain in the chest has almost completely disappeared; the burns have commenced to cicatrise and every thing promised that the patient would soon be able to quit the hospital.

You may say, and with truth, too, that these cases present no special interest; but it is by a report of the treatment of simple accidents, that I can give you the most correct idea of the existing state of French surgery. With all the great points of the science in France, American physicians are of course familiar. With the minor but not the less important features they may not be so well acquainted. Relative to the local treatment of burns, we see that the two surgeons of St.

Louis hospital use widely different therapeutic agents. Thus, Jobert employs, as a general method, whatever may be the degree of the burn, refrigerants; covering the wounded surfaces with a greased piece of linen, upon which he afterwards applies bladders filled with ice. By this method he proposes to obtain, 1st, the mitigation of the acute pain incident to burns; 2d, the diminution or prevention of the consecutive inflammatory reaction; 3d, the diminution of the suppuration which precedes and follows the fall of the eschars; 4th, and finally, the more rapid cicatrisation of the wounds. It is necessary to remark, however, that there are cases in which the employment of ice is contra-indicated; as for instance when the patient presents complication on the part of the thorax, or even a predisposition to pulmonary diseases; when the burns, situated on the chest or the posterior part of the trunk, are of very great extent; in which latter case the dorsal decubitus will be impossible, and in the other case we should aggravate pulmonary affections, or at least favor their development. Finally, ice should not be employed during the period of catamenial discharges. Besides these there are, of course, other circumstances which render the use of ice inadvisable, to which, however, it is unnecessary to allude.

Malgaigne, on the other hand, prefers to the employment of ice the use of the oleo-calcarious liniment and carded cotton. He associates these two means, because the application of the cotton alone does not calm with sufficient promptness the pain, nor the liniment alone protect the injured surface from friction, as of the bed clothes, for instance. After the example of Velpeau, Malgaigne employs a liniment composed of equal parts of olive oil and lime water. M. Miguel, who seems to have occupied himself especially with this subject, prefers the following preparation: oil of sweet almonds, one part; lime water, two parts. Malgaigne, in employing the former liniment and dressing, thinks that he obtains especially, the diminution, and in some cases even the cessation, of the pain. Doubtless there are cases in which ice would be applicable, and probably more efficient than the liniment and carded cotton; and there are other cases, again, where ice could not be used, and the liniment and cotton would be of service. There is no question in my mind that both means possess considerable value.

If I have not given you any of M. Rostan's clinical lectures which he commenced a month or two ago, it is because my private courses occupied me in such a way, that I found it impossible to do so in a satisfactory manner, and not because each one did not contain interesting and valuable matter. The following on the *march of pulmonary phthisis and the cadaveric lesions of this affection*, he delivered a few days since:—In bed No. 11, of ward St. Antoine, we had a woman who had died of a tuberculous affection of the lung. You see so many examples of this disease in the hospital, and you are so familiar with its history, that we shall not insist upon it at any length this morning. Nevertheless, this case presents a particular feature which renders it interesting and worthy of your attention. We proceed to recall, in as few words as possible, its history, and the pathological alterations that were found at the autopsy. The woman was aged twenty-four years. When she arrived in our wards she was, as you remember, very vague in her replies to our questions; we were obliged to interrogate her a long time with great patience and in the most rigorous manner; she replied briefly and unsatisfactorily to the questions addressed to her. Auscultation and percussion sufficed, however, to reveal her pathological state. She was confined, a short time ago—four or five weeks; since which time too she has felt sick; but in a general way, and without being able to indicate any organ which suffered particularly either in its functions or sensibility. She complained of weakness of the inferior members, from which one might infer, and in fact from which we did ourselves infer, that the spinal marrow was affected. Let us remember the symptoms observed in this respect; for although nothing of this kind was found at the autopsy, it is well to bear it in mind. She was of feeble constitution, lymphatic temperament; we pointed out to you the narrowness of the thoracic and abdominal cavities, particularly the first; the thinness and slenderness of the extremities; the paleness of the face. At the time of her entrance and during the following days she had great thirst, dry tongue, no appetite, habitual constipation; her urine normal, and passed with facility. The pulse was 112; respiration 32; slight pains in the breast, vague and without any precise and determined seat. Cough not very frequent; some mucous expectoration without trace of pus. Menstruation performed regularly. The catamenia have appeared once since her confinement. Skin hot; no chilliness. The nervous system seemed unaffected, save the feebleness of the legs which we have mentioned. In this state of things you should percuss and auscultate the chest with care: this is what we did. We found very evident dullness under the left clavicle. Respiration was strong, hard, *soufflant*, almost cavernous. When the patient spoke, resounding of the voice; numerous crepitations during strong inspiration, principally during the cough. We pronounced, as our diagnosis, tuberculous pulmonary affection, at the third period. There was not the slightest question of this, and the physical signs were unfortunately of disheartening clearness. Remark, moreover, that this woman assured us, that before her pregnancy her health had been perfectly good; that it had appeared equally good during gestation, and that before labor she coughed very little, if at all.

The author of a recent work endeavors to establish, that pregnancy and parturition exercise no appreciable influence upon pulmonary tuberculisation. Some distinguished men have asserted that the march of the tuberculous affection was in no respect accelerated by these conditions. But at the very moment when the authors of whom we speak were setting forth this opinion, we have had occasion to observe, in both the hospital, and in our private practice, examples of tuberculous women in whom, contrary to this assertion, the disease had progressed very rapidly after the labor. We must retain the conviction, then, after the daily observation of numerous facts, that under circumstances of this kind, tuberculisation makes ordinarily very rapid progress, and are obliged to dissent from the views above alluded to. The very case about which we are occupied this morning, is in direct opposition to those views. Before and during her pregnancy, the woman had very little cough. She is scarcely delivered when the tuberculous affection assumes a more rapid pace, and in a very little time, only a few weeks, death supervenes. As to the general state, when we saw this woman the first time, we could have said that the disease with which she was affected was *latent*, although the local state was already advanced, as was proved by the dullness, the pectoriloquy, the rudeness, and the *soufflant* character of the respiration. There had been constipation, which is an uncommon circumstance at the period when the alteration is so serious. During the greater part of her sojourn in our wards, the disease seemed to remain stationary. We ought to say that, the diagnosis once established,—the affection recognised in a positive, and perfectly distinct manner,—we did not subject the patient to an attentive daily examination. We contented ourselves with merely a glance at the general condition, and with watching for the appearance of symptoms which should indicate a grave alteration of the economy. One day the patient complained of having a diarrhea. This led us to make an attentive and thorough examination; the febrile state was more intense; in a word, every thing indicated that, arrived at its third degree, the tuberculisation had assumed a more rapid march. The general health suffered, the emaciation became greater, the features of the face became altered, the eyes sank, the cheek bones projected, and were colored with that ominous red; the face generally assumed a livid yellow tint; the general phenomena became more and more clear, and the diarrhea augmented from day to day. Up to this time we confined ourselves to the prescription of a palliative treatment, consisting chiefly of calming drinks. In order to combat the diarrhea, we have had recourse to a very ancient preparation, at one time very much used, but almost fallen into neglect at present; we allude to *diascordium*. This preparation is extolled in the old pharmacopœias as a remedy for chronic diarrhea; it is composed of a great number of substances, the majority of which are astringents, among which we may mention the *rose de provins*, gentian galbanum, snake weed, (polygonum bistorta,) storax, opium, etc., etc. The diarrhea ceased; after many intermissions it reappeared anew, and every time we mastered it by a few doses of the diascordium. We recommend the diascordium to you as being occasionally a precious resource. In certain cases analogous to this, the course consists in treating the symptoms, when we are able to do nothing more rational or efficacious. The patient died a few days since.

After having described in detail what passed during her life, let us pass to an account of what we have found at the autopsy. You know that independently of what we find in the chests of tuberculous persons—that is, apart from the affection itself, the pneumonias, the intercurrent and concomitant pleurisies—there is another very frequent series of lesions, namely, the alterations of the intestines, and especially of the small ones. These alterations are so common, so constant, that they generally receive but little attention. They are the result of an accumulation of tuberculous matter in the submucous cellular tissue; and ulceration succeeds to the softening of this matter. Every one knows that these ulcerations have the greatest resemblance to those of typhoid fever. The case now in hand, in the actual state of the intestine, proves at once how great is this analogy, and at the same time that the identity is not such that it is impossible to distinguish always the one disease from the other. What is the character that may assist us in making this distinction? It is the determination of the seat. If the ulcerations are without the patches of Peyer and the follicles of Brunner, it will be easy to see, at first glance, that they were not produced by typhoid fever. Then the best marked, and deepest ulcerations are not in the ilio-cæcal valve, in phthisis, as they are in typhoid fever. They occupy even the most elevated points of the intestine. This is the most frequent disposition. At other times however, there is a closer resemblance between tuberculous ulcerations—of which you have seen so remarkable an example—and those of typhoid fever, of which we have shown you a specimen within a day or two. In the case in hand, as also in one of typhoid fever, to which we have just made allusion, the ulcerations occupied the patches and follicles. Still in this instance there was no typhus; and yet there cannot be the least doubt as to the nature of the ulcerations. Some authors have pretended, because of the identity of seat and aspect, that it was sometimes impossible to establish, with any certainty, the distinction between the ulceration in the one and the other case; in other words, an intestine being given, to determine accurately whether it belonged to a tuberculous or a typhoid patient. Now we will point out a character, by paying attention to which you may get clear of any doubts that may arise in your minds, and which will preserve you from confounding two pathological conditions so essentially different; this is the examination of the mesenteric ganglia.

In typhoid fever, as in tuberculisation, the mesenteric glanglia are tumefied, voluminous, diseased; but in the first case, the affection is purely inflammatory; they are hypertrophied, red, bluish, slate colored; they sometimes even suppurate. In tuberculous phthisis, they are always white, exclusively formed of tuberculous matter, and perfectly recognisable when divided with the scalpel. They are cheese-like and opaque; that is, they are tubercle, in the full force of the expression. And, now, in the case before us, what are the alterations that we have found in the lungs? Ancient adhesions, flabby lungs, caverns containing softened tuberculous matter mixed with blood; though we cannot decide whether the presence of the blood is not owing to the too violent pressure of the hands of those who made the autopsy. The presence of these caverns in the points which we had marked out, accounts for the cavernous *souffle*, and the gurgling sound heard during life. It is not our intention to give here the anatomo-pathological history of pulmonary phthisis, and shall finish our morning lesson by calling your attention to the fact that these lungs offer the three degrees of tuberculisation—from the miliary granulations, abundantly disseminated throughout the whole of the lung, to the excavations situated as they most generally are, at the superior part.

M. Hugier, in the latter part of April, commenced his annual course of lectures, at Lourcine hospital, on the diseases of the genital organs of the female. One of his lectures was devoted to the diagnostic signs furnished by the inspection of the vulva and of the orifice of the vagina. He commenced by saying—Among the different means that may aid you in the diagnosis of the diseases of the genito-urinary organs of the female, there is one which authors scarcely mention that merits your attention. It is the inspection of the external genital parts. By the aid of this simple and unique means, we are often enabled to recognise certain facts, to distinguish certain uterine diseases, the existence of which is sometimes overlooked, or detected only after long and repeated examinations, insufficient and even inapplicable in many cases. In fact, you meet with women, who from fear, from excess of sensibility, from a feeling of real or affected modesty, refuse to allow you to pass into the interior of their sexual organs either your finger or the speculum. It happens, moreover, that with virgin girls, with women suffering from violent inflammations of the genital organs, or from certain vices of conformation, either natural or acquired, it is impossible to carry our exploration within the vulvar orifice. These, now, are grave and embarrassing circumstances, which render still more precious a diagnosis whose elements are furnished entirely by the inspection of the vulvo-perinæal region in certain cases of uterine affections. The attentive examination of the aspect of the vulvar region conducts us not only to the diagnosis of certain affections of the vulva itself, of the vagina and of the uterus; but enables us also to judge, in some degree, of the morals of women; in certain circumstances of their sexual habits. But this is not the place to expose the various changes that the vulvar and anal regions may present, and to enter into their physiological and moral value. Let us hasten to this simple indication, to this question which possesses a true practical utility; the inspection of the vulvo-perinæal region as applied to the discovery of vulvar, vaginal and uterine diseases. Affections of the vulva, and vagina having been the subject of our lectures last year, we will confine ourselves now to diseases of the uterus. And first, of the aspect of the pudendum in the physiological state, in comparison with the morbid affections of the organs of generation.

1st. *Pregnancy.* The vulva in pregnant women presents a violet tint, barely indicated by authors, and no where described with the proper detail. This tint, of a violet red, entirely characteristic, is not uniform, not equally well marked throughout. Little apparent upon the internal surface of the great lips, it becomes very sensible upon the internal surface of the nymphæ, in the neighborhood of the clitoris and *meatus urinarius*, upon the tubercle of the vagina; when the mucous membrane becomes in some degree more delicate, thinner and more transparent. This violet tint cannot be confounded with the discoloration which certain women present from a varicose disposition of the veins of the genital organs. This latter is inseparable from the course of the venous trunk, whose form and direction it reproduces, whilst the tint of which we are speaking is in no degree confined to the course of the veins; it is spread out, so to speak, like a sheet; the discoloration, besides, does not offer the same shade in these two different states. It is at the end of the second month of pregnancy that we commence to observe it; and it becomes very evident in the course of the third month. The existence of the fact to which we refer, possesses an incontestable value; it precedes all the other sensible diagnostic signs of pregnancy, which can never be recognised before the end of the fourth month of gestation. We believe that the appearances of which we speak do not exist except during pregnancy, as it is only then that they can be demonstrated; a circumstance which acquires a certain value in a medico-legal point of view. As to its origin—as to the causes which produce this violet tint itself, we may remark, that this phenomenon can only be attributed to the state of hyperæmic congestion in which the uterus is found, and to the compression of the pelvi-uterine veins. But is it objected, how does it happen that this tint does not exist in those states where various diseases of the uterus develop, to a considerable degree this organ, and constitute it equally the seat of a hyperæmic congestion? We will not attempt to refute this argument by physiological reasons; what is allowed us, and what is important is the establishment of the pathological fact itself.

Another peculiarity among pregnant women is the state of humidity of the vulva, which under certain circumstances may amount to a true discharge. The cellular tissue of the labia pudendi, nymphæ and clitoris, becomes the seat of a slight serous infiltration. Further, the piliferous follicles become hypertrophied and give to the external surface of the labia a granulous, mammellated appearance. The sudoric apparatus and the sebaceous glands perform their functions with increased activity, which causes an abnormal exaltation; a gluey and sticky condition of the parts. Whereas in those cases that may simulate pregnancy, the labia pudendi and nymphæ in some degree wither, and grow thin.

Let us sum up the characters furnished by the aspect of the vulvar region, which may serve us in the diagnosis of pregnancy. 1st. Violet tint, characteristic, and almost never wanting. 2d. Hypersecretion of the piliferous follicles; hypersecretion of the sebaceous follicles. 3d. Hyper-sudoric secretion; various degrees of œdema.

II. *State of the vulva after delivery.* It is especially in a therapeutic and medico-legal point of view that the examination of the state of the vulva after delivery merits particular attention. Let us see now whether this examination will lead us in any given case to determine whether labor has occurred at a period more or less recent. In a woman lately confined, we observe an abnormal dilatation of the vulvar orifice, a depression and shortening of the perineum, a widening of the fourchette with or without rents, with or without recent cicatrices; the folds of the vulva, and the nymphæ are a little thicker than ordinary; from the vulva there flows a turbid liquid, resembling lees of wine, of an earthy or brick dust color, and specific odor. Relative to the rents of the vulva, you should especially remark their seat, extent and direction. If these rents are the result of labor, they will be seated, by preference, towards the posterior part of the vulva, at the fourchette, then upon the lateral parts, more often to the left; finally towards the union of the nymphæ with the labia majora, and particularly on the left side. You should note the form and direction of these rents. Those that are found in women recently delivered ordinarily offer a triangular form; the summit of which triangle looks towards the centre of the orifice of the vulva, whilst the base is directed towards the thighs; that is to say, from within outwards. Such is not the disposition of the solutions of continuity which are the consequence of external violence, as of the introduction of foreign bodies into the interior of the genital organs. Uniting all the signs that we have alluded to and described, you will be able to conclude as to the existence of an anterior and recent labor, and thus furnish useful light in a question of medical jurisprudence. The aspect of the vulva affords other peculiarities, in certain constitutional and pathological conditions of the female. In blondes —soft and lymphatic women—in chlorotic and scrofulous women, those who have a habitual amenorrhea, the labia pudendi, nymphæ and clitoris are poorly developed; the vulva is covered with very few hairs; the mucous membrane is pale, shining and cold, the papillæ and follicles are but slightly marked; the color of the vulva at its entrance is hardly distinguished from that of the skin of the labia majora and minora, as you will have an opportunity of observing at the end of the lecture, in two women laboring under a uterine affection. The skin of the vulvar region is deprived of the pigmentum that is observed more or less abundantly in *brunes* of good constitution, and which makes the labia pudendi and nymphæ present a very marked difference in color from the abdomen and thighs. We moreover find in lymphatic and chlorotic women the orifice of the vulva and the beginning of the vagina often bathed by vaginal and uterine liquids; in this case you may rest assured that you have to deal with a hypersecretion from the vagina, arising from atony, with a uterine catarrh of the same nature, and that tonics and ferruginous preparations are indicated.

Let us add to the preceding some words upon the character of the liquids which bathe the vulva. If in separating the lips of the vulva you see escape an abundant, reddish, sanguinolent liquid, of a strong odor, you may suspect the existence of a cancer, of a polypus, or of a fibrous body projecting into the cavity of the uterus. If the liquid is creamy, of a yellowish white, more or less consistent, and not thready, nor elastic, it comes from the vagina. If there are albuminous flocculi, if the liquid is tenacious, transparent, of a greenish or yellowish white, it proceeds from the uterus, and you have to do with a simple hypersecretion, or a purulent uterine catarrh. We may still further remark, that in the case of cancer of the uterus, of fibrous tumor, of polypi, the rugæ and the folds which exist upon the mucous membrane of the vulva are almost entirely effaced; the papillæ and follicles have, so to speak, disappeared; the parts assume a smooth aspect, polished and shining, of a dim, pale tint. By this character alone we were able to expose, not long ago, an error of diagnosis in the subject of a woman regarded as having a uterine cancer, but who in reality had only a visico-vaginal fistula. This fistula, in consequence of the sojourn of the urine in the vagina, had inflamed and indurated the parts. Our first diagnosis based only upon the aspect of the vulva, was confirmed by a more profound and complete exploration by means of the touch and the speculum. What we have said must be sufficient to convince you of the value of the examination of the external genital organs in certain diseases of the uterus, some of which we will treat of at our next meeting.

The matter of these last pages will no doubt strike the reader as curious. It exhibits French medicine and French society in what, to many, must appear a singular aspect. No where, probably, but at Paris, would it have entered into the minds of physicians to resort to examinations such as are here described, as a common practice, to make out the diagnosis of common diseases, or to decide upon the existence of pregnancy; and in no other society but that of this capital would females submit to such exposure for such purposes. The subject is instructive. We see to what refinements the processes of diagnosis are carried by practitioners here, and what novel and strange methods are put in requisition. A superficial examination is sufficient to convince us, that in this department of the science the French physicians are taking the lead of all others, and that in the investigation of diseases incident to females, they enjoy facilities such as no other practitioners can command.

PARIS, July, 1847.

